# Calcium store refilling and STIM activation in STIM- and Orai-deficient cell lines

**DOI:** 10.1007/s00424-018-2165-5

**Published:** 2018-06-22

**Authors:** Sisi Zheng, Lijuan Zhou, Guolin Ma, Tian Zhang, Jindou Liu, Jia Li, Nhung T. Nguyen, Xiaoyan Zhang, Wanjie Li, Robert Nwokonko, Yandong Zhou, Fukuan Zhao, Jingguo Liu, Yun Huang, Donald L. Gill, Youjun Wang

**Affiliations:** 1 Beijing Key Laboratory of Gene Resource and Molecular Development, College of Life Sciences, Beijing Normal University, Beijing, 100875 People’s Republic of China; 2 Key Laboratory of Urban Agriculture (North) of Ministry of Agriculture, College of Biological Science and Engineering, Beijing University of Agriculture, Beijing, 102206 People’s Republic of China; 3 Center for Translational Cancer Research, Institute of Biosciences and Technology, College of Medicine, Texas A&M University, Houston, TX 77030 USA; 4 Key Laboratory of Cell Proliferation and Regulation Biology, Ministry of Education, Institute of Cell Biology, College of Life Sciences, Beijing Normal University, Beijing, 100875 China; 5 Department of Cellular and Molecular Physiology, The Pennsylvania State University College of Medicine, Hershey, PA 17033 USA; 6 Institute of Biosciences and Technology, Department of Molecular and Cellular Medicine, Texas A&M University College of Medicine, Houston, TX 77030 USA

**Keywords:** STIM1, Orai1, SOCE, Calcium, Endoplasmic reticulum, 2-APB

## Abstract

Mediated through the combined action of STIM proteins and Orai channels, store-operated Ca^2+^ entry (SOCE) functions ubiquitously among different cell types. The existence of multiple STIM and Orai genes has made it difficult to assign specific roles of each STIM and Orai homolog in mediating Ca^2+^ signals. Using CRISPR/Cas9 gene editing tools, we generated cells with both STIM or all three Orai homologs deleted and directly monitored store Ca^2+^ and Ca^2+^ signals. We found that unstimulated, SOCE null KO cells still retain 50~70% of ER Ca^2+^ stores of wildtype (wt) cells. After brief exposure to store-emptying conditions, acute refilling of ER Ca^2+^ stores was totally blocked in KO cells. However, after 24 h in culture, stores were eventually refilled. Thus, SOCE is critical for immediate refilling of ER Ca^2+^ but is dispensable for the maintenance of long-term ER Ca^2+^ homeostasis. Using the Orai null background triple Orai-KO cells, we examined the plasma membrane translocation properties of a series of truncated STIM1 variants. FRET analysis reveals that, even though PM tethering of STIM1 expedites the activation of STIM1 by facilitating its oligomerization, migration, and accumulation in ER-PM junctions, it is not required for the conformational switch, oligomerization, and clustering of STIM1. Even without overt puncta formation at ER-PM junctions, STIM1_1–491_ and STIM1_1–666_ could still rescue SOCE when expressed in STIM KO cells. Thus, ER-PM trapping and clustering of STIM molecules only facilitates the process of SOCE activation, but is not essential for the activation of Orai channels.

**Electronic supplementary material:**

The online version of this article (10.1007/s00424-018-2165-5) contains supplementary material, which is available to authorized users.

Store-operated Ca^2+^ entry (SOCE) is a ubiquitous Ca^2+^ influx process occurring within junctions between the endoplasmic reticulum (ER) membrane and plasma membrane (PM), brought about by ER-PM interactions of the ER Ca^2+^ sensor stromal interaction proteins (STIM1 and STIM2), and PM-resident Orai Ca^2+^ channels [30, 31]. Triggered by the depletion of ER luminal Ca^2+^ levels, STIM proteins undergo conformational changes [36] that lead to the engaging and opening of Orai1, Orai2, and Orai3 Ca^2+^ channels, also known as Ca^2+^ release-activated Ca^2+^ (CRAC) channels [11, 30]. The SOCE process mediated by various combinations of STIM and Orai homologs is crucial for many types of cell functions [1, 5, 6, 21, 37, 45]. Aberrant STIM-Orai signaling has been linked to a number of human diseases including immunodeficiency, myopathy, atherosclerosis, autoimmune diseases, and cancer [1, 21, 33, 37]. One hurdle that limits our understanding of the role of SOCE in cellular functions is the existence of multiple homologs and splice variants of STIM and Orai. Despite of several gene knockdown or knockout studies carried out in cell culture or whole animals [33], there is still a lack of studies systematically dissecting the cellular roles of SOCE on a clean genetic background. More specifically, even though SOCE has been linked to the refilling of ER Ca^2+^ stores since the introduction of the concept of SOCE more than 30 years ago [32], the exact role of SOCE in the refilling and maintenance of ER Ca^2+^ stores has remained controversial [3, 33].

The sequential steps leading to the functional coupling of STIM1 with Orai1 at ER-PM junctions have thus far been worked out in considerable details [25, 30, 43]. Sensing decrease in ER Ca^2+^ levels, the cytosolic region of STIM1 adopts a more activated conformation, revealing SOAR/CAD (STIM-Orai activating region/CRAC activation domain) and enabling oligomerization and puncta formation at ER-PM junctions, leading to binding and activation of Orai1 Ca^2+^ channels [11, 30, 36]. STIM oligomerization and formation of STIM1 puncta is considered a good indicator of STIM activation [22, 30, 36]. Both the kinetics of STIM oligomerization and factors that affect STIM puncta formation have been studied extensively, and several regulatory factors on ER have been described [36], including SARAF [28], POST [18], Junctate [38], and STIMATE/TMEM110 [15, 34]. A “diffusion-trap” mechanism has been proposed to explain STIM1 puncta formation [30, 43], and subsequent Orai-clustering has been proposed to result from crosslinking of Orai channels by STIM proteins [25, 46]. PM tethering of activated STIM1 via the binding of its poly-basic C-terminal tail to negatively charged phospholipids in the inner leaflet of PM [2, 8, 40] leads to the translocation of STIM1 molecules into ER-PM junctions. The SOAR/CAD domains of these STIM1 molecules then bind, trap, and cluster more Orai1 channels within the same region [20, 29], and the trapped Orai1 channels can cluster more STIM1 molecules [43]. However, exactly how PM tethering through the K-rich region of STIM1 and the interaction with Orai1 channels are involved in the STIM1 activation process is still not understood.

The aim of the current research is to examine the effects of PM tethering of STIM1 to early events of SOCE activation and to better understand the role of SOCE in the maintenance and refilling of ER Ca^2+^ store in a genetically clean background. We first generated STIM or Orai knockout (KO) HEK cells with the CRISPR/Cas9 genome-editing technology [35]. Using these engineered cells together with a recently developed, highly sensitive ER Ca^2+^ indicators CEPIA1er [39], we scrutinized the contribution of SOCE to ER Ca^2+^ homeostasis in HEK cells. We also delineated the relative roles STIM1 K-rich region and Orai binding on PM tethering of STIM1 molecules and defined how each functional element contributes to STIM1 clustering and accumulation at ER-PM junctions during SOCE activation.

## Results and discussion

### SOCE is exclusively mediated by STIM and Orai in HEK cells

**Fig. 1:**
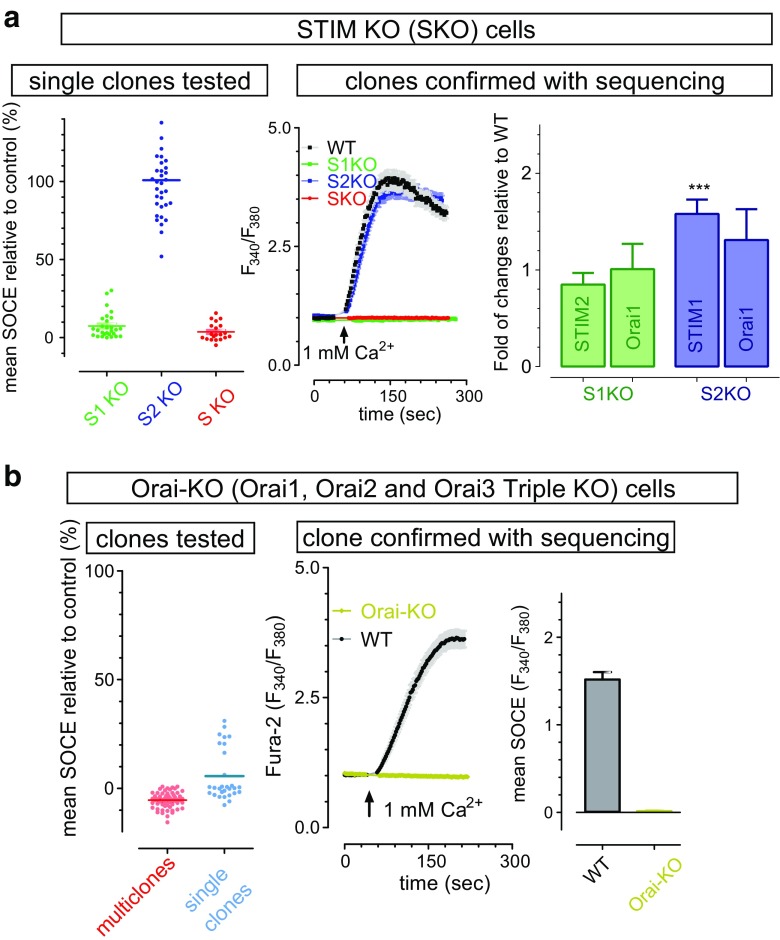
Characterization of HEK STIM or Orai knockout (KO) cells. **a**, **b** TG-induced Ca^2+^ influx (SOCE) in KO cells measured with Fura-2 imaging. Before recordings, ER stores were emptied by 10-min-pretreatment with 1 μM TG. **a** Ca^2+^ responses and mRNA levels of SOCE genes of WT and STIM KO cells. Green, STIM1 (S1KO); blue, STIM2 (S2KO); red, STIM1/2 double KO (SKO). Left, mean SOCE responses of individual clones survived from selection; middle, typical traces of cells from WT or confirmed STIM KO clones mean SOCE: 2.2 ± 0.1; 0.01 ± 0.005; 2.2 ± 0.08; − 0.04 ± 006; for WT, S1K, S2K, and SK, respectively. (*n* = 3); right, statistics showing changes in mRNA levels of SOCE genes in KO cells relative to those in WT cells (*n* = 3, ^***^p < 0.001, WT vs KO, *t* test). **b** Ca^2+^ responses of Orai1/2/3 triple KO (Orai-KO) cells. Black, wild type; light olive, Orai-KO. Left, mean SOCE responses of individual survived clones (blue dots) or individual cells of multi-clonal cells (red dots); left panel, representative traces of TG-induced Ca^2+^ entry in WT and Orai-KO cells; right, statistics of the middle panel. All the data are presented as mean ± SEM

To aid the mechanistic dissection of SOCE in cells with a clean genetic background, we used the CRISPR/Cas9 genomic editing tool to generate single, double, or triple knockout HEK cell lines (Fig. S1). We examined the effect of STIM or Orai-KO on SOCE. The ER Ca^2+^ stores of Fura-2 loaded WT or KO HEK cells were first depleted by 10-min incubation with the SERCA pump blocker, thapsigargin (1 μM), in a nominally Ca^2+^-free solution. One millimolar of Ca^2+^ was subsequently added back to the external solution, and the level of SOCE was measured by the increase in Fura-2 ratio (Fig. 1a, b). We examined HEK cells in which either of the two STIM homologs was genetically deleted. Using STIM1 knockout cells (STIM1-KO or S1KO), we determined that SOCE was reduced by 97% compared to wild type (WT) HEK cells. By contrast, in STIM2-KO (S2KO) cells, the level of SOCE remained largely unaffected, likely due to low expression of STIM2 or some compensatory upregulation of STIM1 expression after STIM2 KO (Fig. 1a, right most panel) or both. In cells with confirmed STIM1-STIM2 double KO (S1/2-KO, SKO), SOCE was completely abolished (Fig. 1a, red). These results indicate that SOCE is primarily mediated by STIM1 in HEK cells, and STIM2 only contributes marginally to SOCE under the extreme conditions. Similarly, using Orai triple knockout cells (Orai1/2/3 knockout, Orai-KO) (Fig. 1b, light olive), there was no Ca^2+^ influx following store depletion. Overall, Ca^2+^ imaging results from either STIM KO or Orai-KO cells clearly demonstrate that STIM and Orai proteins are the essential physiological mediators of SOCE in HEK cells.

### STIM proteins undergo oligomerization to form intracellular clusters without PM tethering

For the first time, we are able to examine molecular determinants that drive STIM oligomerization and puncta formation on an *ORAI* null background using our KO cell lines. In response to store depletion, STIM proteins adopt an activated conformation and oligomerize, then eventually form puncta at ER-PM junctions [30, 36, 43]. The K-rich region and SOAR/CAD domain of STIM1 were shown to be crucial for puncta formation via their interactions with lipids and Orai channels on PM, likely through a diffusion-trap mechanism [30, 43] where oligomerized STIM1 moves freely along ER membrane via Brownian diffusion and directly interact with PM-resident phospholipids [2, 8, 40] and Orai channels [20, 29]. STIM1 proteins are thus accumulated at ER-PM junctions to form puncta [30, 43]. However, it is still unclear whether such diffusion-trap mechanism is essential for driving STIM1 oligomerization and/or puncta formation. We then examined whether STIM1 protein, with its K-rich region deleted, can still form puncta in triple Orai knockout (Orai-KO) cells.

**Fig. 2:**
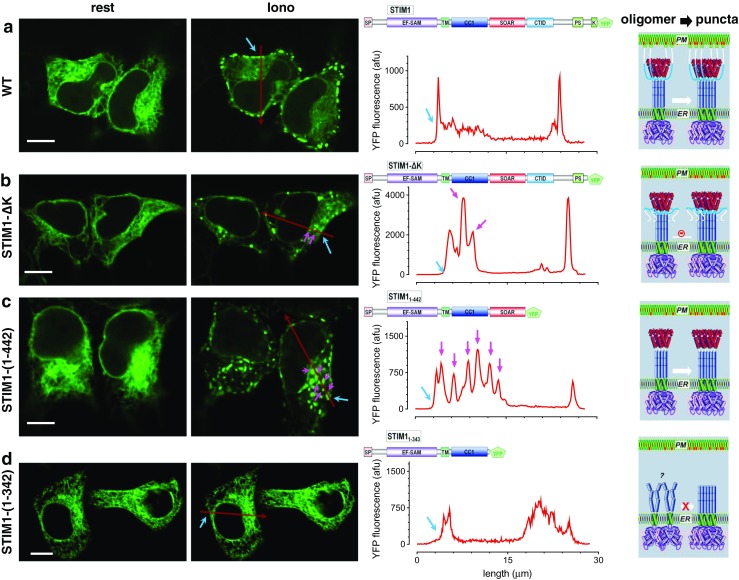
STIM1 protein without K-rich region could still form puncta in HEK Orai-KO cells. Different STIM1 constructs with YFP tagged at their C-terminus were transiently expressed in HEK Orai-KO cells and examined with confocal microscopy. Left, images of the middle plane of typical puncta-forming cells before (rest) and after store depletion (Iono: 5 min after 2.5 μM ionomycin treatments); scale bar, 10 μm; middle, profiles of YFP fluorescence along the red arrows (shown in images on the left) in store-depleted cells at two different focus planes. Red traces, in the middle plane of cells. Cell edges were indicated with blue arrows, and puncta formed outside of ER-PM junctions within cells were indicated with purple arrows. Right, diagrams showing proposed oligomerizing and clustering of STIM1 constructs deep within cells or at ER-PM junctions. **a** Full-length STIM1. STIM1 puncta are mostly localized on the peripheral of the cells. **b** STIM1-ΔK. In all the cells expressing STIM1-ΔK we examined, about 5% of them could form sparse puncta after store depletion. Without the help of PM-anchoring poly-K region, some STIM1 puncta are located within the interior of cells (indicated by purple arrows). **c** STIM1-(1-442). Without the entire region C-terminal to SOAR/CAD, massive STIM1 puncta were formed all over the cell. **d** STIM1-(1-342). After the deletion of SOAR/CAD region, STIM1 lost its ability to form puncta after store depletion. The densities of intracellular clusters that were formed outside of ER-PM junctions were 0, 1.6 ± 0.4, 13.2 ± 3.2, and 0% for wt, STIM1-ΔK, STIM1-(1-442) and STIM1-(1-342), respectively (*n* = 3, at least six puncta-forming cells were examined each time)

We first examined the distribution of full-length WT STIM1-YFP before and after store depletion in Orai-KO HEK cells. Consistent with previous studies carried out in native HEK cells [22, 36], STIM1 clearly aggregated and formed puncta at cell periphery after store depletion (Fig. 2a). The result indicates that Orai proteins are not required for STIM to form puncta at ER-PM junctions. Indeed, this argument is further corroborated by the recent finding that light-induced oligomerization of the STIM1 K-rich region alone is sufficient to trigger STIM1-like puncta formation at ER-PM contact sites [10].

We then deleted the K-rich region (residues 667–685) in STIM1 (STIM1_1–666_) and checked its cellular distribution after store depletion. Similar to previous reports done in WT cells [12, 23, 29, 44], STIM1-ΔK-YFP (STIM1_1–666_-YFP) [12] failed to form puncta in most tested Orai-KO cells. However, STIM1-ΔK did form sparse puncta in about 5% of Orai-KO cells (Fig. 2b). Without Orai channels and its PM-anchoring K-rich domain, the STIM1-ΔK puncta were located far away from cell periphery (Fig. 2b, indicated by purple arrows). We therefore designated this type of puncta as intracellular “clusters” to discriminate it from puncta at the ER-PM junctions. These results show that STIM1-ΔK could still oligomerize to form clusters in a process that does not involve PM tethering of K-rich region of STIM and the PM-resident Orai channels.

To better examine the clustering of STIM molecules, we further deleted the entire region downstream of SOAR (STIM1_1–442_), including the C-terminal inhibitory domain (CTID, residues 470–491) that may inhibit STIM1 clustering [14, 17], and examined the distribution of STIM1_1–442_ in the Orai-KO cells. STIM1_1–442_ formed large intracellular clusters after store depletion (Fig. 2c). As we expected, STIM1 truncation that does not have SOAR/CAD domain and all the downstream regions (STIM1_1–342_) cannot form any clusters or oligomerize after store depletion (Fig. 2d). This result is consistent with a previous study done in WT cells containing endogenous Orai molecules [29]. Taken together, these results clearly indicate that PM tethering of STIM1 by Orai1 or the K-rich region of STIM1 is not required for the oligomerization and clustering of STIM1. Instead, the region of SOAR/CAD plays the critical role in driving STIM1 self-oligomerization.

**Fig. 3:**
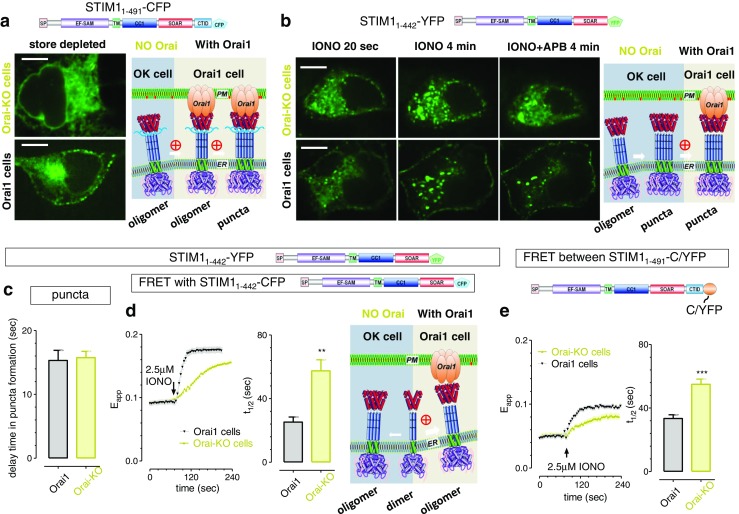
The effects of PM tethering on the oligomerization and clustering of STIM1. ER Ca^2+^ stores were depleted with ionomycin (2.5 μM). **a**, **b** Orai1 promoted the formation of STIM1 puncta at the peripheral of store-depleted HEK cells. Figures on the left are confocal images; figures on the right are diagrams showing the oligomerization and puncta formation within or at ER-PM junctions. Scale bar, 10 μm. **a** Confocal images of STIM1_1–491_-CFP in HEK Orai-KO (top panel) or Orai cells (lower panel) 5 min after store depletion. STIM1_1–491_ can barely form some puncta within Orai-KO cells (top image), while it can form large amount of puncta at the periphery of cells stably overexpressing Orai1 (bottom image). **b** Typical confocal images showing the distribution of STIM1_1–442_-YFP at certain periods of time after addition of ionomycin (IONO) or IONO together with 2-APB (50 μM). In HEK Orai1-his cells, STIM1 puncta eventually got redistributed and trapped to the peripheral of cells. Application of 2-APB can greatly diminish STIM1 puncta within cells. After 2-APB, the densities of intracellular STIM1 clusters were reduced from 15.0 ± 3.4 to 8.1 ± 2.3% in Orai-KO cells and from 7.1 ± 0.8 to 4.8 ± 0.9% in Orai1-his cells. (*n* = 3, at least five cells were examined each time). **c** Statistics showing average delay time for the appearance of ionomycin-induced STIM1_1–442_-YFP. For Orai1-his cells, punctate images were measured at the footprints of cells. Clearly, there is no significant difference in the onset time for the clustering of STIM1_1–442_-YFP (*p* = 0.8, *t* test, three independent repeats, more than 23 cells examined in each group). **d** Effects of ionomycin on FRET signals between STIM1_1–442_-C/YFP transiently expressed in Orai-KO or Orai1 cells. Left, representative traces; middle, statistics; right, diagram showing the effect or Orai1 on trapping and oligomerizing STIM1 at ER-PM junctions. **e** Effects of ionomycin on FRET signals between STIM1_1–491_-C/YFP transiently expressed in Orai-KO or Orai1 cells. Left, typical traces; right, statistics. All the data are presented as mean ± SEM

Nevertheless, activated STIM proteins do need to migrate toward ER-PM junctions to engage and gate Orai channels [30, 38]. Both previous studies performed in WT HEK cells [12, 23], and our own results in Orai-KO cells (Fig. 3 a, b) clearly showed the critical role of the K-rich region in recruiting STIM1 into this unique membrane contact sites formed between the ER membrane and PM. We further examined the contribution of Orai1 channels in recruiting STIM1 into ER-PM junctions by using STIM1 constructs lacking the K-rich region. For STIM1_1–491_-CFP protein that still retained the first lobe of the C-terminal inhibitory domain (CTID) [14], its ability to form puncta at ER-PM junctions after store depletion was greatly impaired in KO cells (Fig. 3a, top image; Fig. 2b). However, in store-depleted cells stably overexpressing Orai1 (Orai1 cells), there were clearly STIM1 puncta at the edge of cells (Fig. 3a, bottom image). For STIM1_1–442_-YFP protein which contained neither K-rich nor the CTID region, intracellular clusters were found all over the cells in both Orai-KO and Orai1 cells (Fig. 3b, left two images; Fig. 2c) following store depletion. The overexpression of Orai1 did not speed up the initial formation of STIM1_1–442_ clusters, as there is no difference in the delay time of STIM1 clustering after the addition of ionomycin (Fig. 3c). Four minutes later, STIM1 clusters were still mainly localized in cytosol in Orai-KO cells, while they got re-organized and recruited to the PM in Orai1-overexpressing cells (Fig. 3b, middle images). Taken together, these results show that, regardless of their clustering condition, the overexpressed Orai1 is able to enhance the translocation of the activated STIM1 molecules into ER-PM junctions, leading to STIM1 puncta formation at the periphery of cells. Thus, in addition to PM-embedded phospholipids [43], Orai channels are also a contributing factor to cluster STIM molecules at ER-PM junctions.

### PM tethering is important for the clustering of STIM1, but not for the activation of SOCE

We next examined whether PM associations of STIM1 molecules could affect the activation steps occurring before clustering. We first measured FRET signals between STIM1_1–442_-CFP and STIM1_1–442_-YFP in HEK Orai-KO cells or HEK-Orai1 cells (HEK cells stably expressing Orai1). The results show ionomycin-induced increase in FRET signals between STIM1_1–442_ pairs were significantly accelerated in HEK-Orai1 cells (Fig. 3d). Similarly, Orai1 also speeded up the ionomycin-induced FRET increase between STIM1_1–491_-CFP and STIM1_1–491_-YFP (Fig. 3e). These results indicate that PM tethering of STIM1 molecules can facilitate STIM1 oligomerization. In agreement with our previous reports done with full-length STIM1 in native HEK cells [42], 2-APB has no effect on FRET signals between STIM1_1–442_ molecules (Fig. S2b, blue trace). Neither did it have any effect on FRET signals between C-terminally tagged STIM1_1–491_ (Fig. S2b, green trace). These results indicate that 2-APB does not affect the cytosolic oligomerization status of STIM1 molecules (Fig. S2d). Even though 2-APB cannot affect the oligomerization status of STIM_1–442_, it can greatly reverse the STIM1_1–442_ puncta formation in Orai-KO cells (Fig. 3b, top row). Similarly, STIM_1–491_ is less capable to form puncta in Orai-KO cells (Fig. 3a, top left image), yet FRET imaging results indicate that they can still oligomerize in Orai-KO cells (Fig. 3e). Thus, these results indicate that the processes of formation of STIM1 oligomers and STIM1 puncta can be separated.

**Fig. 4:**
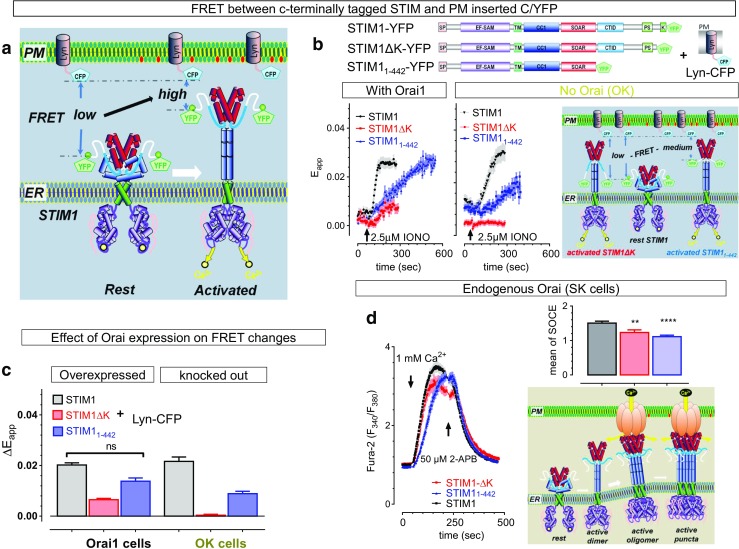
The effects of PM tethering on STIM1 unfolding and its activation of Orai1. **a** Diagram showing the experimental design used to indicate the folding status of the cytosolic region of STIM molecules: the distances of STIM1 constructs with YFP tagged at their C-terminus to PM is monitored by their FRET responses with PM-inserted Lyn-CFP. If the FRET signal is high, then its C-terminus is closer to PM, indicating an extended configuration; and vice versa. **b** Typical FRET responses between PM-inserted Lyn-CFP and STIM1-YFP or STIM1ΔK-YFP in HEK Orai1 cells (left traces), or Orai-KO cells (middle traces). Right panel, diagram showing configurations of activated STIM1 constructs that may explain results in Orai-KO cells. **c** Statistics of results in **b**. Δ*E*_app_ values were obtained by subtracting rest *E*_app_ from the plateau *E*_app_ 4~8 min after ionomycin treatments. Later portion of the traces were not shown in the right panel of **b**, one example of such trace was shown in Fig. S2c. Deletion of K-rich region significantly reduced ionomycin-induced FRET increases in both type of cells (black vs red, *p* < 0.0001, *t* test, *n* = 3), while Orai1 expression have no effect on FRET signals between STIM1-YFP and Lyn-CFP (*p* < 0.47, *t* test). In HEK Orai-KO cells, there is no detectable increase in FRET between STIM1ΔK-YFP and Lyn-CFP. **d** SOCE responses of HEK SKO cells transiently expressing STIM1-YFP, STIM1ΔK-YFP, or STIM1_1–442_-YFP. Before recordings, ER Ca^2+^ stores were emptied by bathing cells in nominally Ca^2+^ free solutions containing 1 μM TG. Ca^2+^ signals are indicated by Fura-2 ratios. Left panel, representative traces; bar graph on the right, statistics; diagram on the right, showing SOCE mediated by interactions between Orai1 channel and oligomerized STIM1-ΔK molecules. All the data are presented as mean ± SEM

We then monitored the unfolding process of different STIM1 mutants using a FRET system. We established a cell line stably expressing PM-inserted Lyn-CFP protein. Whether the cytosolic part of WT or mutated STIM1 molecules is undergoing unfolding is indicated by the changes in FRET signals between PM-inserted Lyn-CFP and various STIM1 constructs with YFP tags at their C-termini (Fig. 4a). We compared the same FRET assay in HEK-Orai-KO cells and HEK-Orai1 cells. When cells overexpress Orai1, ionomycin induced a rapid increase in FRET signals between STIM1-YFP and Lyn-CFP (Fig. 4b, black trace in the left image), thus suggesting a decrease in the distance between the C-terminus of STIM1 and PM-associated CFP and the unfolding of activated STIM1 molecules. The speed and amplitudes of the FRET increases were greatly diminished by the deletion of K-rich regions of STIM (Fig. 4b, red traces in the left image; Fig. 4c), indicating an impairment of STIM1 unfolding. Similar effects were seen in HEK-Orai-KO cells (Fig. 4b, middle images, Fig. 4c). Thus, PM tethering via its K-rich domain substantially accelerated the unfolding, or activation, of STIM1 molecules. After the removal of the other PM tethering factor, Orai channels, the ionomycin-induced increase in FRET between STIM1-YFP and Lyn-CFP was only marginally slowed down (Fig. 4b, c, black), indicating a minor role of Orai channels in expediting the activation of STIM1 molecules. With both PM tethering factors nullified, the ionomycin-induced FRET increases between STIM1_1–442_-YFP and Lyn-CFP was further diminished and slowed down in Orai-KO cells (Fig. 4b, blue trace in the middle figure; Fig. 4c). FRET increases between STIM1-ΔK-YFP and Lyn-CFP completely disappeared (Fig. 4b, red trace in the middle figure) in HEK-Orai-KO cells. These results indicate the critical role of PM tethering and CTID domain in driving efficient STIM1 activation.

However, even with both PM tethering factors abolished, there were still some residual, 2-APB sensitive, ionomycin-induced increase in FRET between STIM1_1–442_-YFP and Lyn-CFP (Fig. S2c). This result indicates that PM tethering is not essential for the unfolding, or activation of STIM1, at least for the STIM1_1–442_ construct. This prompted us to examine the effect of PM tethering on STIM1-mediated Orai1 activation. We thus expressed various STIM1 constructs in STIM1/2 null cells (SKO) and measured the TG-induced SOCE with Fura-2 imaging in these cells. The results showed that all constructs could mediate 2-APB sensitive SOCE in SKO cells (Fig. 4d), indicating some inhibitory effects of 2-APB on SOCE may come from its effects on STIM1 [42]. Taken together, these results clearly demonstrate that, although the poly-basic tail and Orai channels facilitate STIM1 activation, PM tethering of STIM1 molecules is not required for the activation of SOCE.

### SOCE is required for acute ER Ca^2+^ refilling but is dispensable for long-term ER Ca^2+^ homeostasis

Since the proposal of the concept of SOCE by Putney three decades ago [12], there has been a tacit belief that SOCE is critical for the refilling of ER Ca^2+^ stores. Were this true, cells without SOCE following the depletion of STIM or Orai molecules would have severe defects and low viability, as proper Ca^2+^ level within ER is essential for ER function and cell survival. However, transgenic animals with impaired SOCE can still survive (reviewed in [33]), indicating that the SOCE may not be the only source of Ca^2+^ for ER refilling. Our collection of engineered HEK cells depleted of Orai or STIM proteins provides a unique opportunity to re-evaluate this idea with the cleanest genetic background in the same type of mammalian cells.

**Fig. 5:**
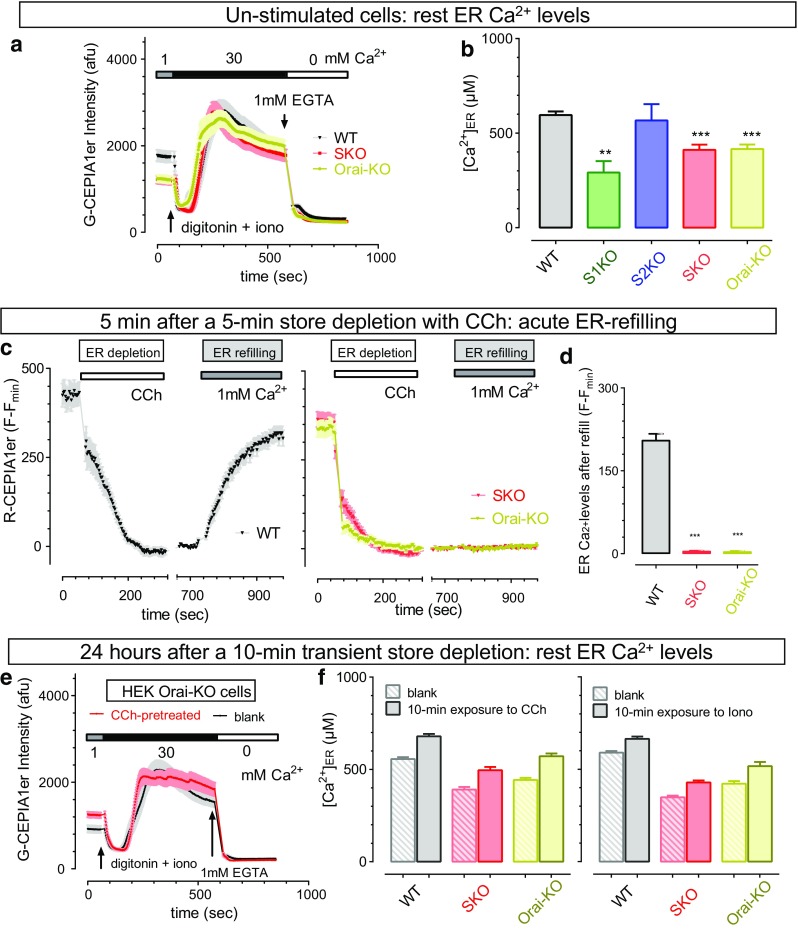
SOCE mediated by STIM-Orai contributes to the refilling and maintenance of ER Ca^2+^ store, but it is not essential for the long-term homeostasis of ER Ca^2+^ levels. ER Ca^2+^ content was measured with G-CEPIA1er or R-CEPIA1er transiently expressed in HEK WT, STIM KO, or Orai-KO cells. **a**, **b** Compared to WT cells, resting ER Ca^2+^ levels in S1KO, SKO, or Orai-KO cells are significantly reduced, while STIM2 KO (S2KO) have no effect on ER Ca^2+^ content. **a** Typical traces showing the entire time course of recordings used to calibrate ER Ca^2+^ levels in HEK WT, SKO, or Orai-KO cells. **b** Statistics showing the effect of STIM or Orai-KO on ER Ca^2+^ levels (*n* > =4, **p < 0.01; ***p < 0.0001; paired *t* test). **c**, **d** As reflected with changes in R-CEPIA1er fluorescence, SOCE process is essential for the acute refilling of ER Ca^2+^ store in HEK cells. In WT cells, after store depletion induced by 100 μM CCh, extracellular addition of Ca^2+^ will result in an increase in Ca^2+^ levels. In HEK SKO or Orai-KO cells that are devoid of SOCE, there is no refilling of ER Ca^2+^ store during the entire period of recording. **c** Typical traces. Left panel, WT cells; right panel, HEK SKO or Orai-KO cells. **d** Statistics. (*n* = 3, *p* < 0.0001, *t* test). **e**, **f** SOCE process is not essential for the long-term maintenance of ER Ca^2+^ levels. After brief store depletion by 10-min incubation in nominally Ca^2+^-free HBSS solution containing 100 μM carbarcol (CCh) or 2.5 μM ionomycin, cells were then cultured in regular DMEM for another day. Afterwards, the ER Ca^2+^ levels indicated by G-CEPIA1er fluorescence were measured and calibrated. **e** Representative traces showing the effect of 10-min CCh-induced store depletion on ER Ca^2+^ levels after 24 h regular culture in HEK SKO cells. **f** Statistic showing 10-min CCh or ionomycin treatments significantly increased the ER Ca^2+^ levels in HEK WT, SKO, or Orai-KO cells 24 h later. (*n* = 4, *p* < 0.0001; paired *t* test). Note: CCh or ionomcyin were then washed away after a 10-min exposure for treatments in **e** and **f**. All the data are presented as mean ± SEM

Using genetically encoded Ca^2+^ indicator (GECI) CEPIA1er that can directly measure ER Ca^2+^ levels with high sensitivity [39], we first examined the effect of SOCE loss on ER Ca^2+^ homeostasis. When measured with transiently expressed G-CEPIA1er (green fluorescence indicator), the resting ER Ca^2+^ concentration was calibrated to be 597 ± 19 μM (*n* = 13) in WT cells (Fig. 5a, b, black) [41]. In cells with minimal (S1KO cells, Fig. 5b, green) or no SOCE (SKO and Orai-KO cells, Fig. 5a, b, red and light olive, respectively), the resting ER Ca^2+^ level was significantly reduced (51.0 ± 10.1, 30.9% ± 4.6, and 30.18 ± 3.99%, respectively) (Fig. 5b). Thus, even though not required for the maintenance of ER Ca^2+^ stores, SOCE indeed contributes to the homeostasis of ER Ca^2+^ stores, but to a lesser extent than one would expect. Consistent with this notion, S2KO cells with regular SOCE (Fig. 1a) have normal ER Ca^2+^ level (Fig. 5b, blue bar). Different from this finding, a previous report using knockdown strategies showed that STIM2 knockdown suppressed TG-induced Ca^2+^ release [3]. One possible explanation for the discrepancy is that some unknown compensatory mechanisms might be induced in S2KO cells with permanent loss of STIM2, but not in cells with transient knockdown of STIM2.

We then examined whether SOCE is required for the acute refilling of ER Ca^2+^ stores (Fig. 5c, d). We depleted ER Ca^2+^ stores by briefly treating cells for 5 min with 100 μM carbacol (CCh), an agonist of muscarinic ACh receptors that could deplete ER Ca^2+^ stores through activation of IP_3_ receptors [9], in nominally Ca^2+^-free solutions (Fig. 5c). After a 5-min break, we added Ca^2+^ back to the external solution and observed a clear Ca^2+^refilling of ER stores in HEK WT cells (Fig. 5c, left). This acute refilling process, however, was completely abolished in SKO (red) or Orai-KO (light olive) HEK cells devoid of SOCE (Fig. 5c, d). These results clearly show that consistent with the initial proposal [32], SOCE is essential for the acute refilling of ER Ca^2+^ stores that occurs approximately within 10 min after store depletion in HEK cells.

Persistent depletion of ER Ca^2+^ stores will induce apoptosis and cause cell death [16, 19]. However, even without SOCE to refill the ER Ca^2+^ stores, SKO or Orai-KO cells can still proliferate properly. This indicates that cells devoid of SOCE may develop alternative strategies to refill their ER Ca^2+^ stores, although their resting ER Ca^2+^ level is lower than in WT cells. We therefore examined whether the resting ER Ca^2+^ levels of HEK SKO or Orai-KO cells could recover from a brief (10 min) store depletion (Fig. 5e, f) after 24 h. To test this, ER Ca^2+^ stores of WT, SKO, or Orai-KO HEK cells expressing G-CEPIA1er were first transiently depleted by 10-min incubation with DMSO (blank controls), CCh, or ionomycin in Ca^2+^-free Hank’s Balanced Salt Solution (HBSS) solutions. After 24 h in DMEM medium, the resting ER Ca^2+^ levels were measured and calibrated [39, 41] again. Surprisingly, even though Orai-KO cells have neither SOCE nor detectable store refilling 10 min after acute store depletion with CCh (Fig. 5c, light olive trace), they showed normal resting ER Ca^2+^ levels 24 h later, which was comparable to WT cells (Fig. 5e, red vs black traces). Similar results were also seen in WT and SKO HEK cells (Fig. 5f, left panel). This effect is independent of reagents used for store depletion, as similar effects were seen in the same cell lines transiently depleted with ionomycin (Fig. 5f, right panel). These findings imply that SOCE is not required for the long-term ER Ca^2+^ homeostasis in HEK cells. It is not clear whether there exists another specific mechanism to refill ER Ca^2+^ store, and further research is needed to address this.

## Concluding comments

One of the well-studied SOCE is a process mediated by a two-component system, STIM and Orai, that undergoes dynamic assembling at the ER-PM junctions. The understanding of the activation process of STIM protein is complicated by the presence of Orai and the PM tethering via its K-rich region. We thus generated Orai triple knockout HEK cells using the CRISPR/Cas9 technology and examined the role of K-rich region of STIM in the activation process. We found that the binding of Orai1 also contribute to this process and that ER-PM trapping of STIM seems to be exclusively mediated by its binding with phospholipids and Orai channels on PM (Fig. S2a). However, our results indicate that PM tethering is not required for the intracellular clustering of STIM1 after store depletion. Further studies are needed to clarify whether activated STIM1 can self-organize into clusters, or it may need the help from other ER-resident factors like SARAF [28], POST [18], Junctate [38], or STIMATE/TMEM110 [15, 34]. Our Ca^2+^ imaging results show that ER-resident STIM1 mutants (STIM1-ΔK or STIM1_1–442_) with diminished PM clustering ability can still induce SOCE, although to a lesser extent. Thus, PM tethering of STIM is not required for SOCE activation, it only facilitates the activation of Orai channels. Indeed, our FRET results show that these two PM tethering factors definitely accelerate the entire activation process of STIM1, ranging from its unfolding and oligomerizing to its puncta formation at the ER-PM junctions. These effects are likely induced by enriching of STIM molecules at ER-PM junctions through diffusion-trap mechanism. We speculate this trapping of STIM-Orai complexes into puncta at the ER-PM junctions may have important modulatory physiological role in native cells. For example, it may lead to the generation of discrete Ca^2+^ hot spots [24], which further induce different types of Ca^2+^ signature responses [33, 36].

In summary, we have established a full collection of STIM or Orai-KO cell lines, which can serve as useful model cellular systems to investigate the STIM/Orai coupling mechanism with clear genetic background. Using these engineered cells, we have some profound findings which drastically advance our understanding of the activation process of STIM1 and the role of SOCE in HEK cells: (1) K-rich region and Orai binding play no role in the initial activation of STIM1 and the following clustering of STIM1 molecules, but can enrich the STIM1 clusters in ER-PM contact sites to facilitate puncta formation. (2) PM tethering of STIM1 is not required for the activation of SOCE, and the CTID domain downstream of SOAR/CAD does affect the unfolding status of STIM1 even when the store is depleted. This indeed suggests the varied efficiency in activating Orai1 between full-length STIM1 and SOAR/CAD. (3) Our data also showed that, as a Ca^2+^ signaling pathway activated by depleting ER Ca^2+^ levels, SOCE is exclusively responsible for the immediate refilling of ER Ca^2+^ stores. But it might play a lesser role in maintaining long-term ER Ca^2+^ homeostasis in HEK cells. Further research is needed to identify other possible crucial factors that are essential for ER Ca^2+^ homeostasis.

## Experimental procedures

### DNA constructs, primers, cell culture, and transfection

pCMV G-CEPIA1er and R-CEIPIA1er were gifts from Masamitsu Iino (Addgene plasmid nos. 58215 and 58216) [39]. To generate Lyn-CFP and Lyn-YFP, Lyn11 N-terminus sequence coding its 11N-terminal amino acid residues [13] was first synthesized and subcloned into pcDNA 3.1 (+) vector between NheI and BamHI sites, and then, the amplified YFP or CFP sequence was inserted into the downstream of pCDNA 3.1-Lyn11 between BamHI and EcoRI sites. To knockout STIM or Orai, sgRNA-directed knockout of STIM1 and ORAI constructs were made by CRISPR/Cas9 genome-editing technology [46] using the online tool (http://crispr.mit.edu) [7] and were inserted into lentiCRISPR v2 vector at the BsmBI site (Addgene plasmid no. 52961). sgRNA sequences and sequencing primers are listed in Table 1.

**Table 1:** Sequences of sgRNAs and primers used for knocking out STIM and Orai

Target	sgRNA sequence (5′ → 3′)	Target CD site	Primers (5′ → 3′)
STIM1	GTATGCGTCCGTCTTGCCCTG	7–27	F: CGGCTGCACTCCCGGGCTCCTGGC R: GGCAACAGAGGTGGCCCAGCCTTA
STIM2	CATGAGCGCCGGGCTATCGC	389–408	F: GTTGCTGGTGCTCGGGCTGCT R: CGGAACCACTAATATCACTAGGTA
Orai1	GTTGCTCACCGCCTCGATGT	459–440	F: AGCTAGGACTGAAGAGTAGT R: CGAAGGGCACCATGATG
Orai2	GGGGTGCATGCGCTCATGCG	413–432	F: GGAGACGCAGTACCAGTA R: AGAAGTCATCCAGTCCTTATG
Orai3	CCAGAGACTGCACCGCTACG	420–439	F: TCTCTACAGTTGAGGTTATGG R: GCTGGACTAAGGGAGGTA

Cell culture and gene transfections were done as previously described [4, 42]. Briefly, all cells were cultured in regular DMEM medium (HyClone) containing 10% FBS (Cleson Scientific), penicillin, and streptomycin (Thermo Scientific) at 37 °C with 5% CO_2_ [42]. Gene transfections were performed by electroporation (Bio-Rad Gene Pulser Xcell system) using 4 mm cuvettes and OPTI-MEM medium [4]. For HEK cells, a 180 V, 25 ms voltage step pulse was used for electroporation; for HeLa cells, an exponential pulse (260 V, 525 μF, 0.5 ml medium) was used instead. Unless specified, experiments were carried out 48 h after transfection.

### Generation of stable knockout (KO) cells

sgRNA plasmids were introduced into cells via electroporation. Two days later, the transfected cells were selected with 40 μg/ml Zeocin (for sgSTIM2 only) or 2 μg/ml puromycin (for all other sgRNAs) for another 4 days. Afterward, cells were cultured in regular medium for about 10 days, and the survived clonal cells were subcultured into 96 well dishes with a density of one cell/well. The resulting single clones were functionally tested with calcium imaging. Healthy clones with uniform responses were chosen for further examination of DNA modifications in targeted genes.

### Confirming disruptions of genes in clonal cells

Genomic DNA was first extracted from selected clones using TIANamp Genomic DNA Kit (cat. no. DP304-02, TIANGEN Biotech) and then amplified with corresponding primers and 2 × Taq Plus PCR MasterMix (cat. no. kt205-02, TIANGEN Biotech) and sent out for Sanger’s sequencing [15]. KO cells with homozygous gene disruption were thus confirmed. For clones with heterozygous gene disruptions, whose sequencing results show double or multiple peaks, the exact DNA sequence within the targeted regions of each allele were further examined with TA cloning and Sanger’s sequencing. Briefly, corresponding PCR products from heterozygous clones were first purified with Universal DNA Purification Kit (cat. no. DP214-02, TIANGEN Biotech) and then ligated into PGM-T Fast vector. Subsequently, the resulting plasmids were transformed into *Novablue* competent cells and subjected to Blue-White Screening. Finally, DNA from white colonies were extracted, amplified, and sequenced with T7/SP6 primers. As shown in Supplemental Fig. 1, only those clones with genes of interest completely or functionally knocked-out were used for further examinations.

### qPCR analysis

Total RNA was isolated from HEK WT, S1K, or S2K cells with MiniBEST Universal RNA Extraction Kit (TaKaRa, cat. no. 9767) following manufacturer’s protocols. Genomic DNA was removed by DNase I (Takara Bio, Inc., Dalian, China, cat. no. 2270A) digestion, and reverse transcription PCR (RT-PCR) was performed (PrimeScript RT Master Mix, TaKaRa, cat. no. RR036A) to obtain the cDNA templates for real-time quantitative PCR. Real-time qPCR was performed using corresponding primers (Table 2) and the QuantStudio 6 Flex Real-Time PCR System (Applied Biosystems, CA, USA) with 2× RealStar Power SYBR Mixture kit (GenStar Biosolutions, Beijing, China, cat. no. A314). Gene expression levels were normalized to those of human GAPDH [15] [27].

**Table 2:** Sequences of primers used for qPCR

Target	Primers (5′→3′)	Sequences
STIM1	Forward	TCCCTTGTCCATGCAGTCCC
	Reverse	GGAATGGGTCAAATCCCTCT
STIM2	Forward	TTGCTGGAGGAGTTGATGACT
	Reverse	CTGCTGCTTCTGGCTAATGA
Orai1	Forward	TGCTCTGCTGGGTCAAGTTC
	Reverse	GACGGCGAAGACGATAAAGAT
GAPDH	Forward	AACTGCTTAGCACCCCTGGC
	Reverse	ATGACCTTGCCCACAGCCTT

### Single-cell intracellular Ca^2+^ measurements

Intracellular Ca^2+^ imaging was performed using a ZEISS observer-A1 microscope equipped with a Lambda DG4 light source and the MetaFluor software (Molecular Devices).

### Measurements of cytosolic Ca^2+^ signals with Fura-2

To load Fura-2 into cells, HEK cells were first kept in the imaging solution (mM): 107 NaCl, 7.2 KCl, 1.2 MgCl2, 11.5 glucose, 20 HEPES-NaOH (pH 7.2) containing 2 μM Fura-2AM for 30 min, then cells were switched to Fura-2AM free imaging solution for another 30 min [25]. Emission fluorescence at 509 nm generated by 340 nm excitation light (*F*_340_) and 380 nm light (*F*_380_) was collected every 2 s, and intracellular cytosolic Ca^2+^ levels are shown as *F*_340_/*F*_380_ ratio.

### Measurements of [Ca^2+^]_ER_ levels with G-CEPIA1er or R-CEPIA1er

Semrock Bright line GFP-1828A-000 or TxRed-A-Basic-000 filter was used to collect corresponding G-CEPIA1er or R-CEPIA1er fluorescence every 2 s. In situ calibration of G-CEPIA1er was performed in calibration solution containing (mM): 10 NaCl, 140 KCl, 1 MgCl_2_, 20 HEPES, 0.025 digitonin, 0.01 ionomycin, (pH 7.2) [39, 41]. During calibration, 30 mM CaCl_2_ was first included in the calibration solution to obtain maximal G-CEPIA1er fluorescence (*F*_max_), then CaCl_2_ in the solution was switched to 1 mM EGTA to get the minimal G-CEPIA1er fluorescence (*F*_min_). ER Ca^2+^ levels were then obtained with the following eq. (39):

$$ {\left[{\mathrm{Ca}}^{2+}\right]}_{\mathrm{ER}}=672\times {\left[\left(F-{F}_{\mathrm{min}}\right)/\left({F}_{\mathrm{max}}-F\right)\right]}^{0.51} $$

All experiments were carried out at room temperature. Traces shown are representative of at least three independent repeats with each including 15–60 single cells.

### Confocal microscopy and puncta analysis

Imaging of STIM1 puncta were taken with a ZEISS LSM510 confocal microscope equipped with × 63 oil objective (NA 1.4), 488 nm laser, controlled by LSM510 software. Sometimes, 3D imaging of STIM1 puncta was also taken with an UltraVIEW VoX spinning disk confocal microscope controlled by Volocity® software (PerkinElmer, Inc.). The resulting images taken at the footprints or equator of cells were exported, and the fluorescence distributions along a typical line across cells were further analyzed and exported using ImageJ software. The resulting data were then plotted with GraphPad Prism5 software. At least three independent experiments were carried out, and representative data were shown.

### FRET measurements

FRET measurements were recorded and analyzed with procedures similar to those described earlier [26]. Briefly, fluorescence imaging experiments were carried out using a ZEISS observer-Z1 microscope equipped with X-Cite® 120-Q (Lumen dynamics) light source, Semrock Bright Line filter sets (CFP (438 ± 12_Ex_/482 ± 16_Em_), YFP (500 ± 12_Ex_/542 ± 13.5_Em_), FRET_raw_ (438 ± 12_Ex_/542 ± 13.5_Em_), × 40 oil objective (N.A. 1.3), and Axiocam 506 mono Camera (Zeiss). This imaging system controlled by Zen Software was used to collect CFP, YFP, and raw FRET images (*F*_CFP_, *F*_YFP_, and *F*_raw_, respectively) every 10 or 5 s. Calibration of bleed through factors across different fluorescence channels and the measurement of system-dependent factor G were done as described earlier [42]. The system-independent apparent FRET efficiency, *E*_app_, was calculated with MATLAB 2014a and plotted with GraphPad Prism5 software. Representative traces of at least three independent experiments are shown as mean ± SEM.

## Electronic supplementary material

ESM 1 (PDF 859 kb)
