# Supplementary material for: Calcium store refilling and STIM activation in STIM- and Orai-deficient cell lines
Source: Pflugers Arch. 2018 Jun 22;470(10):1555–67. doi: 10.1007/s00424-018-2165-5 (PMC6153602; doi:10.1007/s00424-018-2165-5)

Calcium signaling, store refilling and STIM activation in CRAC-deficient mammalian cells

Sisi Zheng[#1,2], Lijuan Zhou[#1], Guolin Ma[#3], Tian Zhang[1], Jindou Liu[1], Jia Li[1], Nhung T. Nguyen[3], Xiaoyan Zhang[4], Wanjie Li[4], Robert Nwokonko[5], Yandong Zhou[5], Fukuan Zhao[2], Jingguo Liu[2], Yubin Zhou[*3] , Donald L. Gill[*5], Youjun Wang[*1]

Supplemental materials

Figure Legends:

Supplemental Figure 1. Generation of STIM or Orai knockout (KO) HEK cell lines using CRISPR/CAS9 gene editing. Representative sequencing results confirming disruptions of STIM or Orai genes. Top panel: raw sequencing traces; middle panel: alignments of genomic sequences; bottom panel: alignment of predicted amino acids sequences of WT and KO Orai protein around the sites of gene disruption. Red letters: mutations; blue letters: insertions; red star: early termination in translation. (A) STIM1 KO (S1KO). (B) STIM1 KO (S2KO). (C-E) Orai1/2/3 triple KO (Orai-KO). (C) Orai1 KO (O1KO). (D) Orai2 KO (O2KO). (E) Orai3 KO (O3KO).

Supplemental Figure 2. Updated model for SOCE activation and 2-APB's effect on activated STIM1 molecules. A) Diagram showing updated model for STIM1 activation. Upon store depletion, STIM1 dimers will release one pair of Ca^{2+} ions, and change from a folded configuration (a) to an extended one (b). The unfolded dimer will further oligomerize (c) and form clusters (e). The unfolding (a-b) and oligomerizing process (b-c) are greatly facilitated by PM association with its K-rich region. Orai1 bindings also contribute to these two steps. Oligomerized STIM1 molecules are enough to bind and activate Orai1 channels. Recruiting more STIM1 and Orai1 into junctional STIM1 punctate region may help provide "hot" spots for Ca^{2+} signaling under native conditions, but this process may not be essential for SOCE activation. B) Addition of 2-APB did not cause a reduction in the FRET signals between $STIM1_{1-442}$-C/YFP pairs (blue trace) or $STIM1_{1-491}$-C/YFP pairs (green trace). C) Typical traces showing the effects of 2-APB on ionomycin-induced FRET signals between PM-inserted Lyn-CFP and $STIM1_{1-442}$-YFP in HEK Orai-KO cells. 2-APB can rapidly reverse the ionomycin-induced increases between Lyn-CFP and $STIM1_{1-442}$-YFP, indicating the activated STIM1 molecules are reversed back to a folded conformation. D) Proposed model of 2-APB's effects on STIM1 molecules.

Supplementary Figure 1

A)

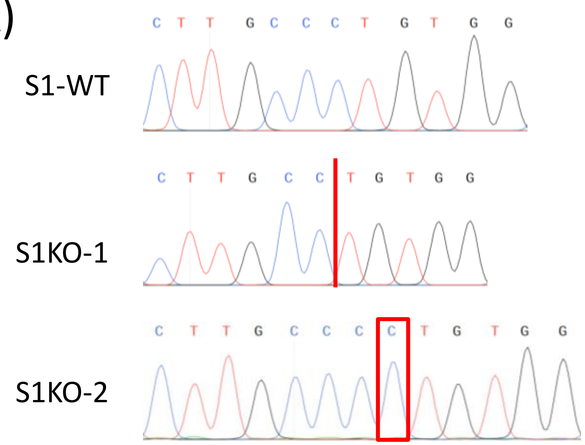

genomic sequence

S1-WT: 19 CTTGCCC-TGTGG 31
S1KO-allele1: 19 CTTGCC—TGTGG 30
S1KO-allele2: 19 CTTGCCCCTGTGG 32

predicted amino acid sequence

S1-WT: 8 ALWLLWGLLLHQGQSLSHSHSEK 30
S1KO-1: 8 APVAPLGTPPAPGPEPQP*---- 25
S1KO-2: 8 ACGSSGDSSCT······N*------ 73

B)

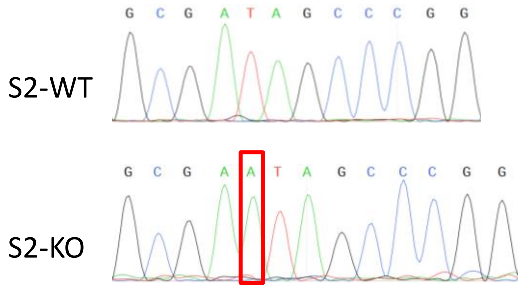

genomic sequence

S2-WT: 389 GCGA-TAGCCCGG 400
S2-KO: 389 GCGAATAGCCCGG 401

predicted amino acid sequence

S2-WT: 117 SAATAASSPAAAAGDSPALMTDPC 140
S2-KO: 117 SAATAASSPAAAAGE*--------- 131

C)

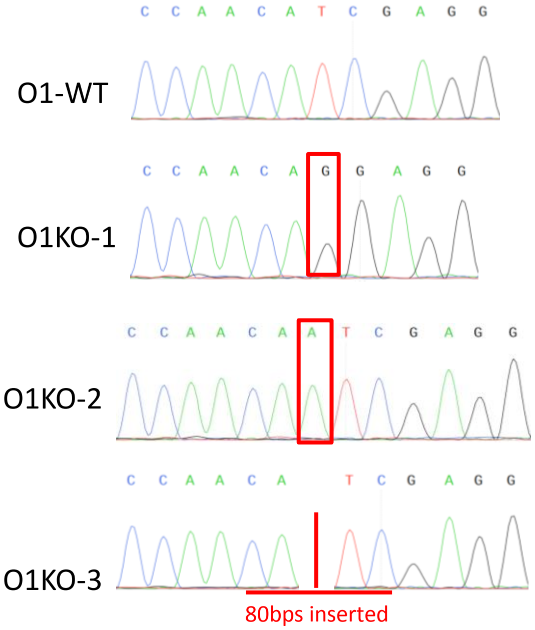

genomic sequence

O1-WT: 437 CCAACA-TCGAGG 448
O1KO-allele1: 437 CCAACAG--GAGG 447
O1KO-allele2: 437 CCAACAATCGAGG 449
O1KO-allele3: 437 CCAACA-TCGAGG 448

TGGGGACTTTCCACACCTGGTTGCTGACTAATTG
AGATGCATGCTTTGCATACTTCTGCCTGCTGGGG
AGCCTGGGGACT

predicted amino acid sequence

O1-WT: 147NIEAVSNVHNLNSVKESPHE 166
O1KO-1: 147NRRR*--------------- 151
O1KO-2: 147NNRGGEQRAQSQLGQGVPP* 165
O1KO-3: 147NMGTFHTWLLTN*-------- 158

D)

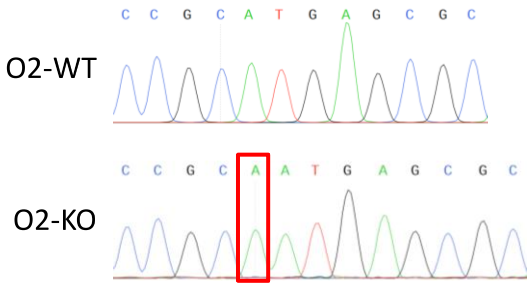

genomic sequence

O2-WT: 412 CCGC-ATGAGCGC 423
O2-KO: 412 CCGCAATGAGCGC 424

predicted amino acid sequence

O2-WT: 137 SPHERMHPYIELAWG 151
O2-KO: 137 SPQ*------------ 139

E)

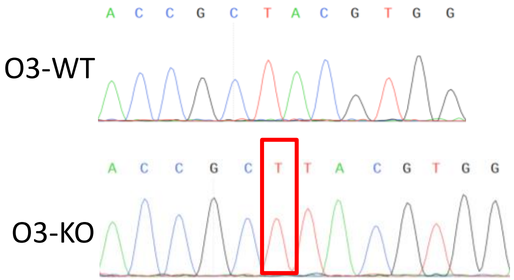

genomic sequence

O3-WT: 431 ACCGC-TACGTGG 442
O3-KO: 431 ACCGCTTACGTGG 443

predicted amino acid sequence

O3-WT: 145RYVELAWGFSTALGTFLFLAE 165
O3-KO: 145RLRGAGLGLLHCPGHLSLPC* 164

Supplementary Figure 2

A) Native cells

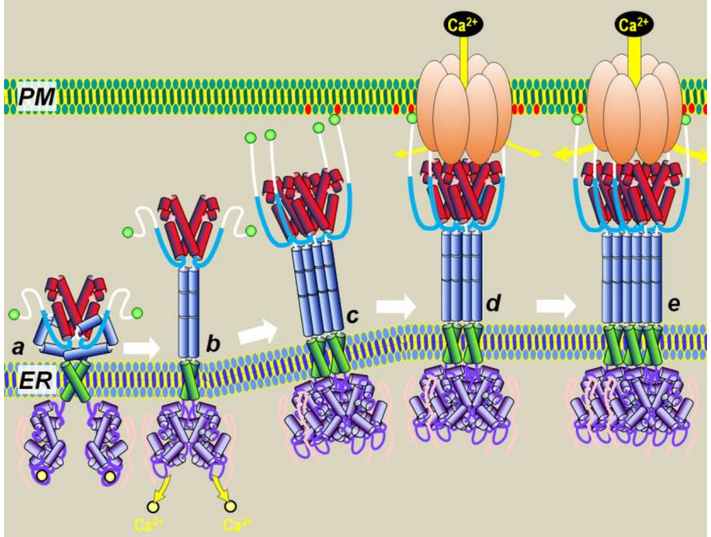

B) FRET between STIM1$_{1\text{-}442}$ or STIM1$_{1\text{-}491}$ in Orai-KO cells

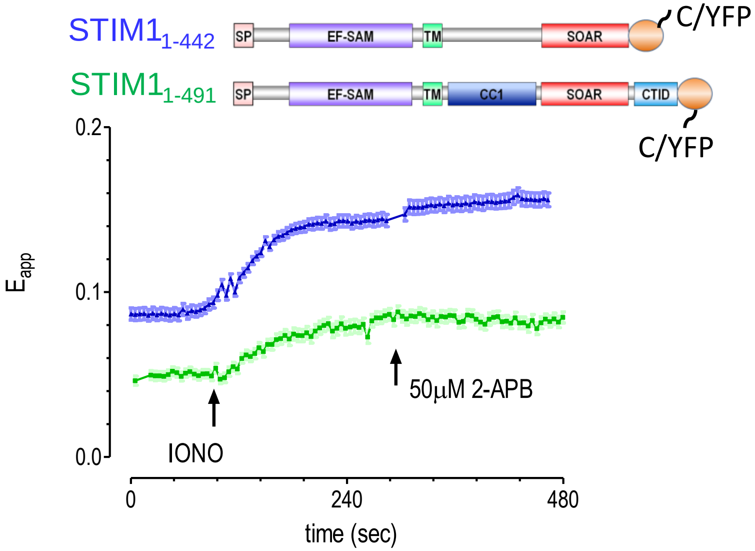

C) Orai knockout (Orai-KO) cells

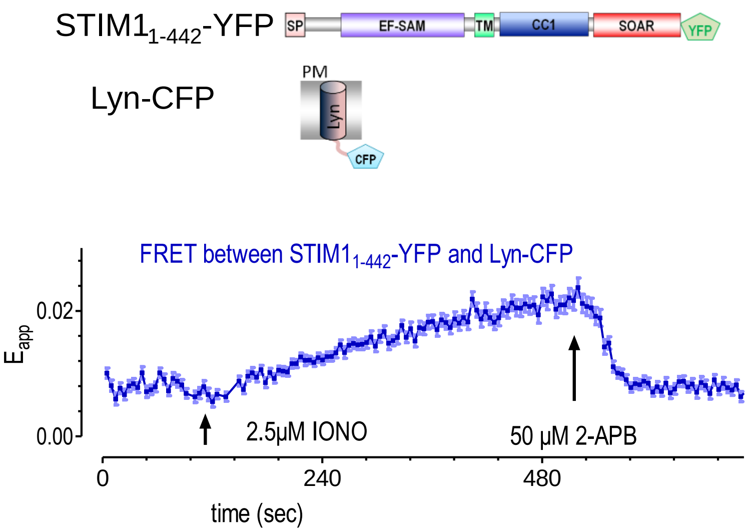

D) Orai knockout (Orai-KO) cells

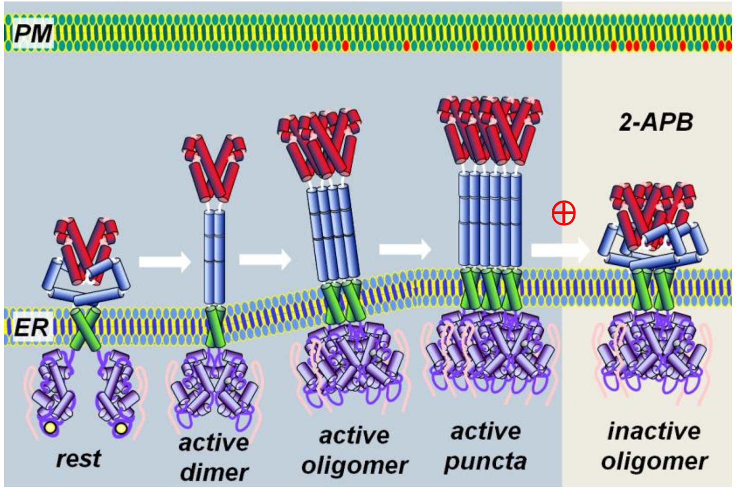

Supplement: Supplementary file 1 — (PDF 859 kb) [file 424_2018_2165_MOESM1_ESM.pdf]
